# Reading Aloud: Discrete Stage(s) Redux

**DOI:** 10.3389/fpsyg.2017.00218

**Published:** 2017-02-27

**Authors:** Serje Robidoux, Derek Besner

**Affiliations:** Cognition and Perception Unit, Department of Psychology, University of Waterloo, WaterlooON, Canada

**Keywords:** reading aloud, discrete stages, cascaded processing, interactive activation, word frequency, stimulus quality, computational models

## Abstract

Interactive activation accounts of processing have had a broad and deep influence on cognitive psychology, particularly so in the context of computational accounts of reading aloud at the single word level. Here we address the issue of whether such a framework can simulate the joint effects of stimulus quality and word frequency (which have been shown to produce both additive and interactive effects depending on the context). We extend previous work on this question by considering an alternative implementation of a stimulus quality manipulation, and the role of interactive activation. Simulations with a version of the Dual Route Cascaded model (a model with interactive activation dynamics along the lexical route) demonstrate that the model is unable to simulate the entire pattern seen in human performance. We discuss how a hybrid interactive activation model that includes some context dependent staged processing could accommodate these data.

## Introduction

In the cognitive psychology of reading there remain several unresolved debates around fundamental issues. Two of these are of particular importance to the major theoretical accounts. One concerns how knowledge is represented in the reading system (distributed vs. localist), while the second concerns how various levels communicate with each other (the processing dynamics). In the present study, we are concerned with the question of processing dynamics in major localist computational accounts of reading aloud.

Various ideas about how information processing unfolds over time have been proposed over the last four decades or so. Discrete stages à la Sternberg were conceptually dominant in the 1960’s and 1970’s (e.g., Sternberg, 1969) and still exert a strong influence on various aspects of human performance (e.g., Sternberg, 1998, 2001). In theories of reading aloud, however, the discrete stages view has given way to the notion of cascaded processing, and then to interactive activation as championed by McClelland (1979, 1987).

Every current major computational model of reading aloud assumes that interactive activation is the primary form of processing dynamics, at least in the lexical system (e.g., Plaut et al., 1996; Coltheart et al., 2001, 2010; Perry et al., 2007, 2010). Here we briefly describe the various proposals for processing dynamics, before turning to an examination of those dynamics in versions of the DRC model that simulate reading aloud (Coltheart et al., 2001, 2010).

### Staged Processing

In the discrete stages approach, processes are ordered serially. Importantly, each stage completes its work before passing the results on to the next stage (hence the descriptor ‘discrete’). This approach allows researchers to use factorial manipulations of variables of interest to identify separable stages: if two factors produce additive effects on mean RT in some task, then one can infer that they influence *separate* stages of processing. If they produce an interaction, then the theorist can infer that they (minimally) affect the same stage of processing (see Sternberg, 1969, 1998).

### Cascaded Processing

McClelland (1979) proposed an alternative way for information to pass through a processing system. In this cascaded account, information is represented as activation in nodes that are used to represent concepts of interest (such as words). Unlike Sternberg’s staged processing, in a cascaded system the processes are no longer discrete. Rather, as soon as any activation is available in one process, that activation flows through to the next process, much like water cascading down a flight of stairs. As the activations in the earlier processes change, so does the flow of activation to the next process. McClelland (1979) demonstrated that such processing dynamics could be used to simulate simple experimental results in memory and location judgements. At first blush, cascaded processing might appear to turn the entire system into a single stage, suggesting that additive effects would be difficult to obtain. However, in an abstract model McClelland (1979) demonstrated that cascaded processing dynamics could produce additivity of factor effects on mean RT provided certain boundary conditions are met, and sometimes even on the variance (but see Roberts and Sternberg, 1993 for some important constraints).

### Interactive Activation

In a cascaded account, activation flows in only one direction through the system, from input to output. McClelland and Rumelhart (1981) expanded this original framework by proposing that activation flows not only forward through the system, but also backward. In their initial model of simple word identification, for example, the presence of the letter F in the first position would send activation forward to words that begin with F. Subsequently, the word FROG would feed activation back to the letter F in the first position, R in the second, etc. They dubbed this back-and-forth process of feedforward and feedback activation flow “interactive activation.” This processing approach has come to dominate computational models of visual word recognition (Plaut et al., 1996; Coltheart et al., 2001; Perry et al., 2007, 2010).

Despite their popularity, interactive activation models have not yet been shown to produce systematic additivity of two factors on mean RT, though this issue has not been widely addressed. To date, only Plaut and Booth (2000) have claimed to produce additive effects in a model with interactive activation processing dynamics. They simulated the additive effects of stimulus quality and word frequency in the context of the lexical decision task using a parallel distributed processing model. This report, if correct, would be important because additivity of these factors has been widely reported in studies with university level readers (e.g., Stanners et al., 1975; Yap et al., 2008 among others). However, Besner et al. (2008) demonstrated that Plaut and Booth’s (2000, 2006) model was highly sensitive to the size of the stimulus quality manipulation (see also Besner and Borowsky, 2006; Borowsky and Besner, 2006 for further observations). That is, when the stimulus quality manipulation was moderate, the joint effects of stimulus quality and word frequency were indeed additive on the proxy for response time in the model. However, when the stimulus quality effect was *smaller*, the joint effects of stimulus quality and word frequency were *under*-additive (a smaller stimulus quality effect for low frequency than for high frequency words; this result has never been reported in the literature to date). With a stronger manipulation, stimulus quality and word frequency had over-additive effects (a larger stimulus quality effect for low than high frequency words). In the lexical decision literature with university level readers, additivity of these two factors is found throughout a wide range of stimulus quality manipulations (e.g., see Yap et al., 2008). To date then, there is no evidence that an interactive activation model can simulate systematic additivity of factor effects.

Related work with a localist computational model that includes interactive activation dynamics along the lexical route is the starting point of the present investigation. Reading aloud is the target task of interest here for several reasons. One is that there is a rich literature with skilled readers at the single word level. Another is the large amount of computational work devoted to reading aloud at the single item level (e.g., see Seidenberg and McClelland, 1989; Besner et al., 1990; Plaut et al., 1996; Coltheart et al., 2001, 2010; Roberts et al., 2003; Perry et al., 2007, 2010; Adelman et al., 2014). We examine a localist dual route computational model here because it is highly successful, it has been implemented in an easily runnable form, and it now provides a more theoretically plausible way of simulating the effect of stimulus quality (more on this later).

### Dual Route Models of Reading Aloud

**FIGURE 1:**
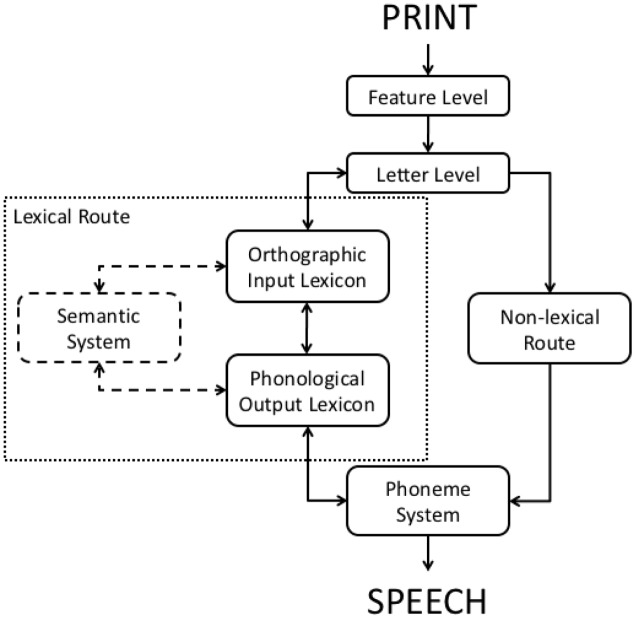
**The general structure of localist, dual route models of reading.** The semantic system is not implemented in any current computational version of a dual route model. Double-headed arrows between levels indicate interactive activation, while single-headed arrows indicate cascaded processing.

Dual route localist models are a class of implemented computational models with a lexical architecture and interactive activation dynamics that have been highly successful at simulating various benchmarks in reading aloud. **Figure 1** depicts the general structure of these models.

The feature level operates in parallel across a letter string, and cascaded activation feeds the letter level. In turn, letter level activation cascades to two separate pathways. The non-lexical pathway (on the right in **Figure 1**) translates the letter string into phonology in a semi-serial, left-to-right fashion. There are currently two different approaches to this process: Coltheart et al.’s (2001) DRC model proposes a set of pre-specified rules for converting print to sound sub-lexically, whereas the CDP+ and CDP++ models (Perry et al., 2007, 2010) use a trained neural network.

The letter level also feeds activation to the lexical pathway (on the left in **Figure 1**), which stores representations for all known words. This route is essentially identical for all implementations of this class of model. The Orthographic Input Lexicon and Phonological Output Lexicon each contain a single localist representation (a lexical entry) for each word known to the model. The Orthographic Input Lexicon represents orthographic (spelling) information, while the Phonological Output Lexicon represents phonological (pronunciation) information. The letter level, Orthographic Input Lexicon, and Phonological Output Lexicon are all engaged in interactive activation (activation feeds both forward and backward through the lexical system as indicated by double-headed arrows in **Figure 1**). Both the non-lexical pathway and the lexical pathway (via the Phonological Output Lexicon) feed activation into the Phoneme System. The Phoneme System holds phonemes for speech output in a buffer that in turn activates articulatory processes (not represented in the models). Note that the Phoneme System and Phonological Output Lexicon are also engaged in interactive activation, while processing through the non-lexical route is purely feed forward cascaded.

### The Joint Effects of Stimulus Quality and Word Frequency

This dual route class of localist models has been very successful in that it correctly simulates a host of experimental findings in the reading aloud task when RT is the main dependent measure (central among them, the effect of word frequency, which accounts for more variance in monosyllabic reading times than any other factor). The models are also able to simulate various forms of acquired dyslexia when the main dependent measure is accuracy (Coltheart et al., 2001, 2010).

Here we focus on one well-established pattern in the reading literature: the factorial combination of word frequency and stimulus quality. Skilled readers are faster to read aloud high frequency words than low frequency ones (e.g., Forster and Chambers, 1973 among many others). An important finding is that when only words appear in the list then the effects of word frequency and stimulus quality interact: low stimulus quality affects low frequency words more than high frequency words (O’Malley et al., 2007; O’Malley and Besner, 2008). This pattern contrasts with that observed when participants perform a lexical decision task rather than reading aloud. In that case, stimulus quality and word frequency are additive so that the effect of stimulus quality is equal for both high and low frequency words (Stanners et al., 1975; Becker and Killion, 1977; Norris, 1984; Wilding, 1988; Borowsky and Besner, 1993; Balota and Abrams, 1995; Plourde and Besner, 1997; O’Malley et al., 2007; Yap and Balota, 2007). This apparent discrepancy between the two tasks was resolved by O’Malley and Besner (2008) who showed that the interaction between stimulus quality and frequency present when reading aloud disappears when non-words are included in the list of items to be read. That is, it is not the task, but the presence of non-words that made the two factors additive in both the lexical decision and reading aloud tasks (O’Malley and Besner, 2008; relatedly see Besner et al., 2010).

### Cascaded Processing vs. Interactive Activation

It might be intuited that an interactive activation model should always produce interactions between two factors, but (a) intuition is a not a substitute for what a simulation actually produces, and (b) at least one cascaded model (feed-forward only) has been shown to be able to produce additive effects of two factors on mean RT provided certain boundary conditions are respected (McClelland, 1979). The central point is that, *a priori*, one hypothesis is that these two factors (stimulus quality and word frequency) produce additive effects when interactive activation is *not* in play, but an interaction when it is. Reynolds and Besner (2004) investigated just this issue in the context of the DRC model. They found an interaction between stimulus quality and word frequency regardless of whether processing in the model consisted of interactive activation, or consisted only of feed-forward cascaded processing.

### Where Does Stimulus Quality Affect Processing?

Nonetheless, we have a reservation about the way in which Reynolds and Besner implemented the stimulus quality manipulation in their study. Stimulus quality is assumed to have its effects very early in processing in the reading system. Consequently, Reynolds and Besner simulated stimulus quality by manipulating the strength of the connections from the feature level to the letter level – the earliest parameter that could be modified. To simulate reduced stimulus quality conditions, they weakened these connections. However, implicit in this approach is an assumption that stimulus quality manipulations do not influence feature processing. A more plausible way to simulate the effect of stimulus quality is to have it influence the input to the feature level itself. This wasn’t possible at the time because, following McClelland and Rumelhart (1981), activation in the feature level nodes of the 2001 DRC model were fixed to either 0 or 1 according to the presence or absence of each feature in the presented letter strings. In essence, the feature level behaved like a discrete stage preceding the rest of the system.

There now exists a version of the DRC model in which the modeler can directly manipulate the strength of the input to the feature level (Coltheart, personal communication, August 23, 2015). This allows us to vary the rate at which activation accrues at the feature level, which better matches how stimulus quality is thought to affect processing in human readers. We therefore use this new version of the front end of the DRC model to address questions about the role that feedback plays in interactive activation models, in particular with respect to simulation of the joint effects of stimulus quality and word frequency. It further addresses whether the results reported by Reynolds and Besner (2004) are specific to their manipulation of stimulus quality, or if such results are also seen in a model with a more plausible manipulation. Given the results of O’Malley and Besner (2008), the key issue is not whether or not stimulus quality and word frequency interact, but rather under which conditions do they interact, and under which conditions are they additive. Ideally, it would be possible to produce both patterns, since both patterns have been observed in the skilled reading literature. To anticipate the results, there is no evidence that the presence/absence of feedback has any impact when the effect of stimulus quality originates at the feature level, as evidenced by the absence of a three-way interaction between stimulus quality, word frequency, and the presence or absence of feedback. When the stimulus quality manipulation is between the letter and orthographic levels as in Reynolds and Besner (2004), we replicate their finding that stimulus quality and word frequency interact regardless of whether or not feedback operates, but note that the *strength* of the interaction is significantly affected by presence or absence of interactive activation. When the manipulation of stimulus quality is moved earlier in the system, we find that the interactions are dampened, but that much of that dampening can be attributed to a general reduction in the effect of stimulus quality manipulations. That is, since the stimulus quality effect is smaller, so are the interactions.

Most generally, these results make it difficult to square some of the experimental results (in particular, additive effects of stimulus quality and word frequency) with the class of localist dual route accounts noted here. The General Discussion provides a way forward in that we propose cascaded processing and staged processing each have a role to play, depending on the context.

## Materials and Methods

In the following simulations we use a version of DRC (2.0.0, beta) in which a new parameter has been added to better simulate input to the reading system. When presented with a word (e.g., FROG), each feature that is present in each letter position receives activation from an external input signal (Feature External Input). This signal can be thought of as the cascading of information from the visual system and other pre-reading visual processes, into the reading system. This new structure means that activation for presented features builds from 0 to 1 over multiple cycles, rather than being simply clamped to the maximum value of 1.0 on the first cycle. As a result, activation throughout the system accrues more slowly than in previous versions of the DRC. The rate at which this activation builds is controlled by the Feature External Input parameter, which is set to 1.0 by default reflecting normal or “clear” stimulus quality conditions.

### Materials

In order to best compare our results with those seen in the literature, we used the word set from O’Malley and Besner (2008) who reported an interaction between word frequency and stimulus quality when only words appeared in the list, and additivity of these two factors when non-words were randomly intermixed with those same words. In their study, the two patterns were observed using a single word list, eliminating concerns that the difference may be due to list effects.

### Ensuring Model Accuracy

We first examined the accuracy of the default model (with interactive activation) to determine how resilient it is to reductions in the quality of the stimulus. The O’Malley and Besner corpus contains 70 high frequency and 68 low frequency words known to the DRC. We tested these 138 items with the model by varying the stimulus quality from 1 to 100% of the default value, using both stimulus quality manipulations independently (input to the feature level, and connections between the feature and letter levels). With these items, the model accuracy was highly resilient to such reductions in stimulus quality, regardless of the location of the stimulus quality manipulation. Using the Reynolds and Besner (2004) approach of reducing the connection strength between the feature and letter levels, the model made no errors until the quality was reduced to 37% of the default strength, where it made a single error. When the locus of the manipulation was moved to the feature level, the model remained perfectly accurate until the stimulus was degraded to 24% of the default weights. For the remaining analysis, we will consider the model’s performance for stimulus qualities as low as 20% of the default value. To ensure that all simulation analyses are based on the same set of items, we remove the single item that was incorrectly named at some of the lower levels of stimulus quality (WAX) from all analyses.

These simulations are concerned with two factors that may affect the interaction between stimulus quality and word frequency in a version of the DRC model of reading aloud (Coltheart et al., 2001). Those factors are the presence or absence of interactive activation (McClelland and Rumelhart, 1981), and the locus of the stimulus quality manipulation (between the feature and letter levels vs. in the input to the feature level). To test these influences, we submitted the O’Malley and Besner (2008) corpus of high and low frequency words to the DRC model.^1^ In each run of the corpus through the model we orthogonally varied the presence or absence of interactive activation, the location of the stimulus quality manipulation, and the strength of the stimulus quality manipulation (by varying the “low stimulus quality” condition through 20, 40, 60, and 80% of the default setting. The default value, or 100%, was always used for the high stimulus quality condition).

The raw simulation data and the analysis scripts (for R) for this project are available at https://osf.io/xutma/.

### Simulating Stimulus Quality

Using a new version of DRC, we examine two alternative ways of manipulating stimulus quality in the model. Until recently, the earliest parameter that was under the control of the modeler was the strength of the connections from the feature level to the letter level. This approach implicitly assumes that that feature processing is not affected by the quality of the stimulus, a clearly untenable view. In this latest version of the DRC, it is possible to weaken the input to the feature level, which can be thought of as allowing stimulus quality to affect the earliest visual and pre-reading processes. Here we examine each of these manipulations separately.

### Simulating the Presence or Absence of Interactive Activation

To examine the influence of interactive activation on the interaction between stimulus quality and word frequency, we submitted the O’Malley and Besner word set to two versions of the DRC 2.0.0 beta. One version is the default model with interactive activation operational throughout the lexical route. In the other version, we eliminated all of the between level feedback along the lexical route of DRC 2.0.0 beta (by setting those parameters to 0).

### Analysis Methodology

For each combination of the presence/absence of interactive activation, and the two locations of the stimulus quality manipulation, we fit the resulting simulation reaction times to a linear mixed effects model with random intercepts for individual words. The general form of the model (using R notation) was as follows:

RT∼SQ*WF+(1|word)

where RT is reaction time, SQ is stimulus quality, and WF is word frequency.

All five levels of stimulus quality (20, 40, 60, 80, and 100% of default parameter values) were included in the same model as a categorical variable. We then developed appropriate contrasts to test for main effect of word frequency, the simple effect of stimulus quality for the high frequency items, and the interaction between word frequency and stimulus quality, for each pairing of the high stimulus quality (100%) with each of the four lower stimulus quality settings. This use of contrasts is intended to ensure the data analysis and reporting match the studies with human subjects, which always used a 2 × 2 factorial design. **Tables 1**–**4** summarize these results for the different processing dynamics and locations of the stimulus quality manipulation.

**Table 1:** Contrasts for simulations *with interactive activation* and stimulus quality manipulated *between the feature and letter levels* (as in Reynolds and Besner, 2004).

Contrast	Estimate	*SE*	df	*t*	*p*
**Low SQ = 80% of default**					
Word frequency	-5.56	1.97	135.85	-2.83	0.0054
Stimulus quality (HF words)	-2.44	0.20	540	-12.09	<0.0001
Interaction (SQ × WF)	0.27	0.14	540	1.91	0.0563
**Low SQ = 60% of default**					
Word frequency	-5.97	1.97	135.85	-3.03	0.0029
Stimulus quality (HF words)	-5.75	0.20	540	-28.47	<0.0001
Interaction (SQ × WF)	0.68	0.14	540	4.75	<0.0001
**Low SQ = 40% of default**					
Word frequency	-6.77	1.97	135.85	-3.44	0.0008
Stimulus quality (HF words)	-11.07	0.20	540	-54.83	<0.0001
Interaction (SQ × WF)	1.48	0.14	540	10.39	<0.0001
**Low SQ = 20% of default**					
Word frequency	-9.02	1.97	135.85	-4.58	<0.0001
Stimulus quality (HF words)	-22.57	0.20	540	-111.78	<0.0001
Interaction (SQ × WF)	3.73	0.14	540	26.20	<0.0001


*SQ, stimulus quality; WF, word frequency (HF, high frequency).*

**Table 2:** Contrasts for simulations with *no interactive activation* and stimulus quality manipulated *between the feature and letter levels*.

Contrast	Estimate	*SE*	df	*t*	*p*
**Low SQ = 80% of default**					
Word frequency	-6.18	0.94	144.6	-6.58	<0.0001
Stimulus quality (HF words)	-3.51	0.32	540	-11.09	<0.0001
Interaction (SQ × WF)	0.37	0.22	540	1.67	0.0954
**Low SQ = 60% of default**					
Word frequency	-6.75	0.94	144.6	-7.19	<0.0001
Stimulus quality (HF words)	-8.78	0.32	540	-27.69	<0.0001
Interaction (SQ × WF)	0.94	0.22	540	4.23	<0.0001
**Low SQ = 40% of default**					
Word frequency	-7.85	0.94	144.6	-8.35	<0.0001
Stimulus quality (HF words)	-17.28	0.32	540	-54.50	<0.0001
Interaction (SQ × WF)	2.04	0.22	540	9.14	<0.0001
**Low SQ = 20% of default**					
Word frequency	-6.18	0.94	144.6	-6.58	<0.0001
Stimulus quality (HF words)	-3.51	0.32	540	-11.09	<0.0001
Interaction (SQ × WF)	0.37	0.22	540	1.67	0.0954


*SQ, stimulus quality; WF, word frequency (HF, high frequency).*

**Table 3:** Contrasts for simulations *with interactive activation* and stimulus quality manipulated *in the input to the feature level*.

Contrast	Estimate	*SE*	df	*t*	*p*
**Low SQ = 80% of default**					
Word frequency	-5.29	2.05	135.29	-2.58	0.0109
Stimulus quality (HF words)	-1.01	0.12	540	-8.25	<0.0001
Interaction (SQ × WF)	-0.00011	0.087	540	-0.00	0.9990
**Low SQ = 60% of default**					
Word frequency	-5.32	2.05	135.29	-2.60	0.0104
Stimulus quality (HF words)	-2.57	0.12	540	-20.93	<0.0001
Interaction (SQ × WF)	0.032	0.087	540	0.37	0.7113
**Low SQ = 40% of default**					
Word frequency	-5.55	2.05	135.29	-2.71	0.0077
Stimulus quality (HF words)	-4.99	0.12	540	-40.55	<0.0001
Interaction (SQ × WF)	0.25	0.087	540	2.93	0.0035
**Low SQ = 20% of default**					
Word frequency	-6.00	2.05	135.29	-2.93	0.0040
Stimulus quality (HF words)	-10.49	0.12	540	-85.28	<0.0001
Interaction (SQ × WF)	0.71	0.087	540	8.16	<0.0001


*SQ, stimulus quality; WF, word frequency (HF, high frequency).*

**Table 4:** Contrasts for simulations with *no interactive activation* and stimulus quality manipulated *in the input to the feature level*.

Contrast	Estimate	*SE*	df	*t*	*p*
**Low SQ = 80% of default**					
Word frequency	-5.88	1.11	135.35	-5.30	<0.0001
Stimulus quality (HF words)	-1.03	0.073	540	-14.08	<0.0001
Interaction (SQ × WF)	0.072	0.052	540	1.40	0.1613
**Low SQ = 60% of default**					
Word frequency	-5.91	1.11	135.35	-5.33	<0.0001
Stimulus quality (HF words)	-2.65	0.073	540	-36.20	<0.0001
Interaction (SQ × WF)	0.10	0.052	540	2.02	0.0440
**Low SQ = 40% of default**					
Word frequency	-6.09	1.11	135.35	-5.49	<0.0001
Stimulus quality (HF words)	-5.49	0.073	540	-75.02	<0.0001
Interaction (SQ × WF)	0.29	0.052	540	5.56	<0.0001
**Low SQ = 20% of default**					
Word frequency	-6.50	1.11	135.35	-5.86	<0.0001
Stimulus quality (HF words)	-12.09	0.073	540	-165.33	<0.0001
Interaction (SQ × WF)	0.69	0.052	540	13.35	<0.0001


*SQ, stimulus quality; WF, word frequency (HF, high frequency).*

### The Role of Interactive Activation

To test for the role of interactive activation, we directly examined the three-way interaction between stimulus quality, word frequency and the presence or absence of interactive activation in two models, reporting separate interactions for each of the stimulus quality locations. The general model was as follows:

RT∼SQ*WF*IA+(1|word)

where IA refers to the presence or absence of interactive activation.

Here again, we constructed appropriate contrasts to evaluate the three-way interaction for the pairing of high stimulus quality (100%) with each of the four lower levels of stimulus quality. **Tables 5**, **6** summarize those contrasts for the two manipulation locations. For brevity, we do not report the lower order terms of the model (main effects and two-way interactions), but the interested reader can replicate our analyses and examine the full results using the data and analysis scripts at https://osf.io/xutma/.

**Table 5:** Interaction of stimulus quality, word frequency, and presence/absence of interactive activation for varying levels of stimulus quality manipulation between the feature and letter levels.

Contrast	Estimate	*SE*	df	*t*	*p*
SQ × WF × IA (low SQ 20%)	0.67	0.33	1215	2.05	0.0411
SQ × WF × IA (low SQ 40%)	0.28	0.33	1215	0.86	0.3904
SQ × WF × IA (low SQ 60%)	0.13	0.33	1215	0.41	0.6831
SQ × WF × IA (low SQ 80%)	0.050	0.33	1215	0.15	0.8778


*SQ, stimulus quality; WF, word frequency; IA, interactive activation.*

**Table 6:** Interaction of stimulus quality, word frequency, and presence/absence of interactive activation for varying levels of stimulus quality manipulation in the input to the feature level.

Contrast	Estimate	*SE*	df	*t*	*p*
SQ × WF × IA (low SQ 20%)	-0.0094	0.27	1215	-0.04	0.9718
SQ × WF × IA (low SQ 40%)	0.016	0.27	1215	0.06	0.9513
SQ × WF × IA (low SQ 60%)	0.036	0.27	1215	0.14	0.8927
SQ × WF × IA (low SQ 80%)	0.036	0.27	1215	0.14	0.8921


*SQ, stimulus quality; WF, word frequency; IA, interactive activation.*

## Results and Discussion

**FIGURE 2:**
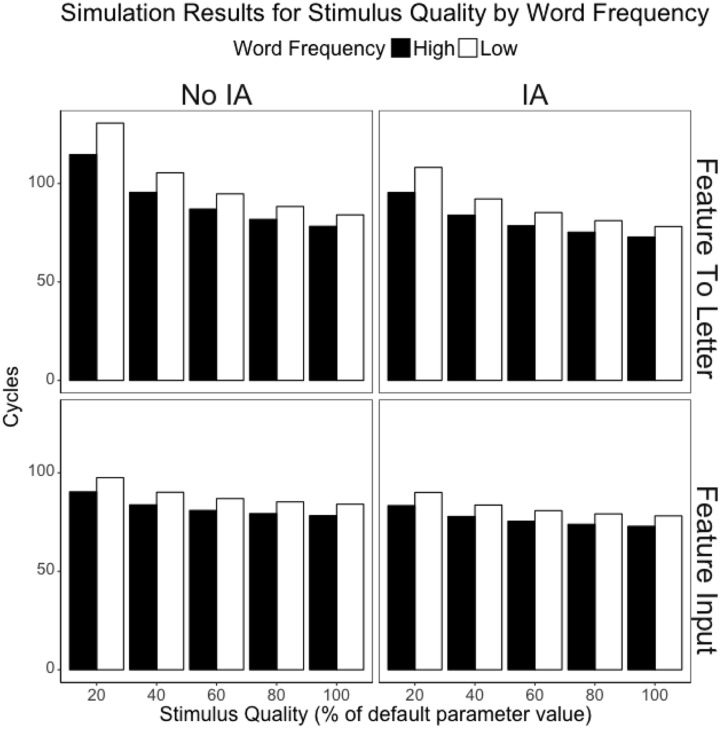
**DRC 2.0.0 beta cycle times for the stimulus quality by word frequency interaction for several manipulations of stimulus quality (from the strongest 20% manipulation to the weakest 80% manipulation), with and without feedback in the lexical route (columns), and varying the location of the stimulus quality manipulation (rows).** 100% represents the clear condition (or high stimulus quality).

**FIGURE 3:**
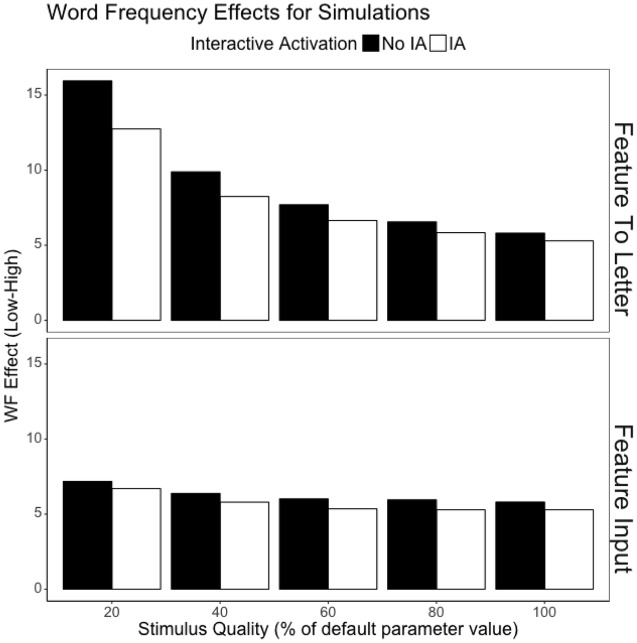
**DRC 2.0.0 beta word frequency effects (low word frequency minus high word frequency) at varying levels of stimulus quality, both with and without interactive activation.** Top panel depicts the results when stimulus quality is manipulated after the feature level (per Reynolds and Besner, 2004), while the bottom panel depicts the results when stimulus quality is manipulated in the input to the feature level.

**Figure 2** depicts the mean cycle times by word frequency, stimulus quality, presence or absence of interactive activation, and the locus of the stimulus quality manipulation. Since here we are concerned with two way interactions (stimulus quality by word frequency), three-way interactions (stimulus quality, word frequency, and the presence or absence of interactive activation), and the four-way interaction of stimulus quality, word frequency, presence/absence of interactive activation, and the locus of the stimulus quality manipulation, we also include **Figure 3**, which depicts the word frequency effect (Low frequency minus High frequency) by stimulus quality, presence or absence of interactive activation, and the locus of the stimulus quality manipulation. Several interesting observations emerge from these simulations.

First, the pattern observed when interactive activation is operating and stimulus quality is manipulated between the feature and letter levels replicates the pattern observed with the original DRC model in Reynolds and Besner (2004): there is a pronounced interaction between stimulus quality and word frequency for all but the weakest stimulus quality manipulation (stimulus quality at 20%: *p* < 0.0001; 40%: *p* < 0.0001; 60%: *p* < 0.0001; 80%: *p* = 0.0563). This contrasts with when the manipulation of stimulus quality is moved to the input to the feature level: the interaction only emerges for the stronger manipulations of stimulus quality (stimulus quality at 20%: *p* < 0.0001; 40%: *p* = 0.0035; 60%: *p* = 0.7113; 80%: *p* = 0.9990). However, it is worth noting that this apparent reduction in the interaction is partly an artifact of another change – moving the manipulation earlier in the system also depresses the influence of the stimulus quality manipulation. When stimulus quality is manipulated in the input to the feature level, the simple effect of stimulus quality for high frequency items is roughly half of the effect observed when stimulus quality is manipulated later in the system (i.e., between the feature and letter levels).

As for the role of interactive activation in the interaction between stimulus quality and word frequency, removing feedback did nothing to the qualitative pattern for either model: interactions remained interactions and additivity remained additivity. Further, the presence or absence of interactive activation only moderated the size of interaction between stimulus quality and word frequency with the strongest manipulation of stimulus quality implemented between the feature and letter levels (*p* = 0.0411). Though it is significantly smaller when interactive activation is absent, the interaction between word frequency and stimulus quality remained significant in both cases (interactive activation: *p* < 0.0001; No interactive activation: *p* < 0.0001). In no other case did the three-way interaction between stimulus quality, word frequency, and interactive activation emerge statistically (all *p*s > 0.39).

If the best way to simulate stimulus quality is by having activation increment continuously at the feature level, then *interactive activation* – a feature that many researchers (e.g., McClelland and Rumelhart, 1981; McClelland, 1987; Coltheart et al., 2001; Patterson and Plaut, 2009 among many others) hold as elemental to cognitive modeling – makes no contribution to performance at all, at least in this context.

### General Discussion

Given McClelland’s (1979) seminal work, it is known that at least one variant of a cascade model can produce additive effects as well as an interaction of two factors on RT. Consequently, it was unclear a priori what the outcome of the present simulations would be. Critically, both the feed forward cascaded version of the model and the version with feedback produced what is seen in the human data when only words appear in the experiment: an interaction between stimulus quality and word frequency.

The remaining problem concerns another pattern seen in O’Malley and Besner (2008). They reported that, indeed, word frequency and stimulus quality interacted, but they also found that these same factors yielded additive effects on RT when *words were intermixed with non-words* (see also Besner et al., 2010, for related findings). O’Malley and Besner proposed that when non-words are intermixed with words, subjects (unconsciously) switch from some form of cascaded processing (either feed forward only, or interactive activation) to processing where at least one process is staged (discrete). This discrete stage prevents stimulus quality from affecting the process that produces a word frequency effect (minimally, the output of the last process to be affected by stimulus quality is staged so that the effect of stimulus quality is not passed on to later processes affected by word frequency).

To be sure, the simulations reported here do provide evidence for both additive effects and interactive effects of stimulus quality and word frequency when the locus of the stimulus quality manipulation is in the input to the feature level. In this case, smaller manipulations of stimulus quality (down to 60% of full quality) produced additive effects whereas stronger manipulations (reduced to 40 or 20%) produced an interaction. Thus, one might suppose that all that is needed in order to see both patterns is that the stimulus quality effect be smaller when additivity is observed than when an interaction is observed with human readers. However, the data reported by O’Malley and Besner (2008) are inconsistent with this account. In their data (see **Table 7**), stimulus quality and word frequency produced an interaction when only words appeared in the list. When words and non-words are intermixed, the same set of words showed clear additivity of stimulus quality and word frequency, *despite no difference in the magnitude of the stimulus quality effect (indexed by the stimulus quality effect for high frequency words) as a function of the presence/absence of non-words in the list.* The bottom line is that it is not possible to simulate both of those patterns (presence and absence of an interaction between word frequency and stimulus quality) for the same set of words using either location for the stimulus quality manipulation tested here (pre-feature-, or post-feature-level).

**Table 7:** The joint effects of stimulus quality and word frequency as a function of context (words only vs. words and non-words) (from O’Malley and Besner, 2008).

	High SQ	Low SQ	SQ effect
**Words only**			
Low frequency	481	602	121
High frequency	470	576	106
*Word frequency effect*	*11*	*26*	
*Interaction*			*15*
**Words and non-words**			
Low frequency	513	624	111
High frequency	506	614	108
*Word frequency effect*	*7*	*10*	
*Interaction*			*3*
(Non-words)	562	677	


*Critically, the presence and absence of an interaction are obtained with no change to the size of the stimulus quality effect for high frequency words. SQ, stimulus quality.*

#### Non-lexical Processing

One possibility for producing additive effects might arise from the purely forward cascaded nature of processing in the non-lexical route (see **Figure 1**).^2^ This line of reasoning would argue that since purely forward cascaded models are known to be capable of producing additivity (McClelland, 1979), perhaps when non-words are present in a word list, the non-lexical route becomes more responsible for reading aloud all items, including words. This approach would require whatever changes are made to respect the conditions that produced additivity in McClelland (1979): reducing the rate of activation in two different processes that are faster than the other processes in the network. Indeed, Ziegler et al. (2009) attempted to simulate additivity of stimulus quality and word frequency in the CDP+ model by adopting precisely this strategy. However, a closer inspection of the Ziegler et al. (2009) data by Besner and O’Malley (2009) reveals that their model was grossly impaired in terms of accuracy for words that do not respect the typical letter-sound correspondences (exception or irregular words, such as PINT). Human readers do not show this pattern in the O’Malley and Besner (2008) data set. In short, the Ziegler et al. (2009) attempt to simulate additivity fails.

An account that relied on stronger non-lexical influences would also make several other predictions including smaller frequency effects since the non-lexical system is not sensitive to word frequency;^3^ generally slower processing of words since the non-lexical system is thought to be slower than the lexical system; and a letter length effect for words since the non-lexical system processes letter strings in a semi-serial left-to-right fashion; and a much greater difficulty with words that do not respect the typical letter-sound correspondences (exception or irregular words, such as PINT), since those rely entirely on lexical processing to access the correct phonology.

#### On the Need for a Hybrid Model

In light of these problems with alternative accounts, we are unable to imagine one or more parameter changes to the DRC model that could be plausibly invoked and shown to produce additive effects of these two factors. Clearly, it is important that both patterns (interaction/additivity) be simulated. We therefore propose that the best account to date is one in which processing undergoes a qualitative change from cascaded to one in which at least one process is staged in the context described here. That is, interactive activation (or only feedforward cascaded processing) is sufficient to simulate the interaction of stimulus quality and word frequency when no non-words are present in the study. In contrast, when non-words are randomly intermixed with words, then at least one process is staged. Elsewhere, it has been argued that this staged processing could be intended to prevent lexicalizations given that non-words are degraded 50% of the time. Related arguments have been advanced in order to explain the triple interaction between stimulus quality, regularity, and the presence/absence of non-words in the list (Besner et al., 2010).

## Conclusion

To be sure, it is unlikely that the field at large will embrace such an account, given that (a) many (indeed, perhaps most) psycholinguists are resistant to the idea of discrete processes, particularly so in the context of reading aloud, and (b) such a hybrid account complicates matters considerably. In addition to invoking qualitatively different ways in which processing unfolds, presumably one or more modules are called for that evaluate the context (detecting the presence of non-words, in this case) and responses that subjects are making, and implement the changes needed in order to optimize performance. Nevertheless, until a cascaded/interactive activation computational model is advanced that can simulate both patterns, we submit that a hybrid account such as suggested here and in O’Malley and Besner (2008; see also Besner et al., 2010; Besner and Risko, 2016) is the best account to date.

Relatedly, we submit that at least one discrete stage is also necessary to account for the additivity of stimulus quality and word frequency that has been widely reported in the context of lexical decision (as noted earlier, see the exchanges between Plaut and Booth, 2000, 2006, vs. Besner and Borowsky, 2006; Borowsky and Besner, 2006; Besner et al., 2008). This additivity was first reported over 40 years ago, and then multiple times since then (e.g., Stanners et al., 1975; Yap et al., 2008). Interactive activation has become the dominant framework for language processing (and in other domains as well) but we submit that it has yet to come to terms with data that predates it, and continues to be reported.

## Author Contributions

This was a close collaborative piece of work. SR did the simulations and, along with DB, wrote the paper.

## Conflict of Interest Statement

The authors declare that the research was conducted in the absence of any commercial or financial relationships that could be construed as a potential conflict of interest.

## Footnotes

1 Trials in the DRC are entirely independent of each other, meaning that all items are read in exactly the same way regardless of which items appeared on previous trials. In other words, DRC is entirely unaware of any contextual differences between conditions. Rather the modeler changes the parameters to suit different contexts. As a result, we do not present the non-words from O’Malley and Besner (2008), since they are meant only to change the context for the words.

2 We thank an anonymous reviewer for suggesting this line of reasoning.

3 One might be tempted to see the frequency effects in **Table 7** (where the effect is 7 ms with non-words present, but 11 ms without) as evidence for this. However, in the full set of experiments in O’Malley and Besner (2008), they reported frequency effects that ranged from 7 to 23 ms when non-words were present. A similar argument can be made for the speed of processing prediction.
